# Varsity medical ethics debate 2018: constant health monitoring - the advance of technology into healthcare

**DOI:** 10.1186/s13010-018-0065-0

**Published:** 2018-09-03

**Authors:** Chris Gilmartin, Edward H. Arbe-Barnes, Michael Diamond, Sasha Fretwell, Euan McGivern, Myrto Vlazaki, Limeng Zhu

**Affiliations:** 1 Gonville and Caius College, University of Cambridge, Trinity Street, Cambridge, CB2 1TA UK; 2 Magdalen College, University of Oxford, High Street, Oxford, OX1 4AU UK; 3 St Peter’s College, University of Oxford, New Inn Hall Street, Oxford, OX1 2DL UK; 4 Trinity College, University of Oxford, Broad Street, Oxford, OX1 3BH UK; 5 Oriel College, University of Oxford, Oriel Square, Oxford, OX1 4EW UK; 6 Newnham College, University of Cambridge, Sidgwick Avenue, Cambridge, CB3 9DF UK; 7 Girton College, University of Cambridge, Huntingdon Road, Cambridge, CB3 0JG UK

**Keywords:** mHealth, Wearable device, App, Patient medical record, Technology, Healthcare

## Abstract

The 2018 Varsity Medical Ethics debate convened upon the motion: “This house believes that the constant monitoring of our health does more harm than good”. This annual debate between students from the Universities of Oxford and Cambridge is now in its tenth year. This year’s debate was hosted at the Oxford Union on 8th of February 2018, with Oxford winning for the Opposition, and was the catalyst for the collation and expansion of ideas in this paper.

New technological devices have the potential to enhance patient autonomy, improve patient safety, simplify the management of chronic diseases, increase connectivity between patients and healthcare professionals and assist individuals to make lifestyle changes to improve their health. However, these are pitted against an encroachment of technology medicalising the individual and home, an exacerbation of health inequalities, a risk to the security of patient data, an alteration of the doctor-patient relationship dynamic and an infringement on individual self-identity. This paper will draw upon and develop these concepts, while contending arguments for and against constant health monitoring. This is not a review of medical devices and health monitoring, but a reflective development and more detailed elaboration of the main points highlighted in the 2018 Varsity Medical Ethics debate.

## Introduction

Technological breakthroughs have far reaching impacts across society, with their benefits also permeating into medicine. An exciting current development is the utilisation of smart phones in clinical practice, with this termed mobile health (mHealth). Simultaneously, devices continuously measuring health parameters are being developed. Devices including those for continuous glucose monitoring (CGM) are now being recommended by the UK National Institute for Health and Clinical Excellence (NICE), in certain circumstances [1]. Constant health monitoring is already entering medical practice, making discussion of its ethical implications highly relevant.

This paper will discuss personal health monitoring (PHM), which refers to “any electronic device or system that monitors and records data about a health-related aspect of a person’s life outside a hospital setting” [2]. This will include devices used for diagnosis, specifically the AliveCor KardiaMobile®, devices monitoring disease states, including CGM, and devices used for contraception, of which we will focus on the app Natural Cycles®. Additionally, we will discuss mHealth apps, such as MyCOPD®, systems providing access to patient medical records (PMRs) and wearable devices monitoring heart rate and calorie usage, such as Fitbit® products, especially as these latter three are more ubiquitous as a result of less stringent regulation. As these technologies are highly varied, each shall be discussed in turn, with the arguments in support and against their use proposed.

### Devices used for diagnosis

Personal health monitoring devices may be used by patients to self-diagnose conditions, one example being the AliveCor KardiaMobile®, a pocket-sized electrocardiogram (ECG) recorder. One of its proclaimed utilities is screening for atrial fibrillation (AF). The UK is reportedly poor at diagnosing AF. The British Journal of General Practice highlighted the diagnosis gap in AF in 2016, with the diagnosed prevalence being 1.6% in England while the modelled estimates put real prevalence at 2.4% [3]. This leaves a third of people with AF undiagnosed. It is estimated that one in five strokes are a result of AF, while the anticoagulant treatment available for AF could reduce the risk of ischaemic stroke by about 70% [3]. Clearly there is much to be desired here. The AliveCor KardiaMobile® aims to reduce this diagnosis gap. It does have impressive recorded sensitivity and specificity results [4] and an Australian study even suggested that the device would be cost effective overall [5].

However, the Australian study does have one critical shortcoming as highlighted by a NICE Medtech innovation briefing [6]- it failed to compare the cost effectiveness of the device to what could be achieved through clinicians, or patients themselves, manually palpating pulses more regularly [6]. As the first stage of AF identification is through feeling an irregular pulse, the requirement for an expensive monitoring device is questionable; simply checking one’s pulse could do the same role. This is the major drawback of such devices designed for patient self-diagnosis: they are expensive tools performing largely unrequired roles. Their use in clinical practice will depend on them passing trials regarding their safety, efficacy and cost effectiveness, similar to other medical interventions.

### Devices monitoring disease states

Continuously monitoring devices may simplify disease management for patients. Taking diabetes as an example, patients are required to adjust their insulin dose according to their exercise levels, food intake and if they show any signs of infection. CGM removes the requirement for patients to repeatedly pin-prick, making monitoring less burdensome. CGM can have a more critical role in notifying patients when their blood sugars are at dangerous levels, potentially averting hypoglycaemic attacks which may progress to coma and death, a powerful safety feature. Additionally, devices continuously monitoring disease may engage patients more in their treatment, enhancing patient self-care and health.

On a broader analysis however, devices that constantly record health parameters may result in patients developing increased anxiety through noting slight non-pathological deviations from their baselines. This hypothesis that constantly recording health parameters may increase patient anxiety lacks validating evidence however, and was not the case in a prospective randomised trial on CGM [7]. The phenomenon of white coat hypertension, an increase in blood pressure when recorded in the clinic, is counteracted by general practitioners offering home kits for measuring blood pressure, so personal monitoring devices are in fact used to resolve health-anxiety in this case.

As monitoring devices become more common place, the consequences they will have on the social environment of patients must be sufficiently accounted for. PHM results in greater medicalisation of the home and personal life of individuals, with the term medicalisation referring to the encroachment of the clinical sphere into matters non-medical. In this case, medicalisation is a consequence of individuals acquiring additional responsibility for their healthcare and being required to utilise these medical devices constantly, including in the home environment [2]. This intrusion into the private life of a patient, so critical for self-identity, must not be so readily dismissed.

### Devices used for contraception

A more radical use of monitoring devices is perhaps when they are used in place of existing medical interventions. This shift is occurring with contraception. Contraception is far from a one-size-fits-all, with each woman’s menstrual cycle individual and hormonal contraception affecting this in equally idiosyncratic ways. Natural Cycles®, a fertility tracking app, uses an algorithm involving the woman’s temperature to estimate whether or not she is fertile and gives advice on whether or not she may need to use barrier contraception. It holds the benefits of having no side-effects and requiring no devices to be implanted. It has gained approval from the European Medicines Agency as a contraceptive with the efficacy rate for perfect use of 99%, and 93% for typical use [8]. This compares to only 91% as a combined statistic for typical use of the oral contraceptive combined pill and progestin-only pills [9].

Whilst Natural Cycles should be applauded for testing efficacy and safety within the established regulatory framework, its use as a contraceptive should still be seen in context. Long-acting reversible contraceptives, such as the coil, are still more efficacious and oral contraceptive pills are more effective when used perfectly with a failure rate of only 0.3% [9]. A concern about the app is that it markets itself as a product, similar to condom brands, unlike many other methods of contraception which are accessible only through healthcare professionals. For individuals to be able to make an informed decision about their contraceptive use, there ought to be clear guidance readily available about each contraceptive option, and relevant healthcare professionals should be well researched on novel devices used for contraception to be able to provide women with accurate advice.

### mHealth apps

Constant monitoring of health has clear benefits in the management of chronic diseases, such as chronic obstructive pulmonary disorder (COPD), asthma and insulin-dependent diabetes. Patients with such diseases are required to self-monitor their condition and carry out their treatment regimen daily, being reviewed by a health professional on an infrequent basis. mHealth apps can reduce the mental workload associated with managing chronic diseases by collating patients’ health information into one place, acting as patient-centred hubs of information. Apps containing medication diaries, education pages and symptom trackers have been created and NHS endorsed, with myCOPD® [10, 11] one such As well as containing these facilities, this app includes videos on pulmonary rehabilitation and inhaler technique enabling patients to seek clear, reliable help on any queries or concerns as they arise.

mHealth may also facilitate a quicker patient response to any deviations from their health baseline through promoting patient self-monitoring, with evidence suggesting that self-monitoring reduces the rates of hospital admissions [12]. Using the example of COPD, when patients with severe COPD notice increased breathlessness or green sputum production, they are required to commence ‘rescue packs’ including antibiotics with steroids. This targets an infection as it develops, preventing too great a deterioration and promoting a faster recovery. If mHealth apps enhanced self-monitoring, they may result in these treatment regimens being started more timely, further improving patient care.

There is a risk however that mHealth may exacerbate health inequalities. Some people from lower socioeconomic groups may not be able to afford a mobile phone capable of supporting the app’s function, therefore a shift towards the uptake of mHealth would be excluding this population group [13]. Such apps also disadvantage those who are less tech-savvy, typically the elderly, lacking the skills required to navigate the app in a productive manner. Detailed and simple tutorials would be a necessity, as well as simple user interfaces, to alleviate this issue.

### Telehealth

Schemes whereby monitoring results are sent to specialists have already been created and have been coined ‘telehealth’. One study analysed the effect of telehealth on patients with COPD, heart failure and diabetes, where patients recorded observations such as blood pressure and blood glucose in their home [14]. This rather simplistic intervention was associated with lower mortality and emergency admission rates [14]. It may also give the patient comfort and peace of mind to know their safety is increased through constant monitoring from healthcare professionals [2].

A concern with such schemes is that there must be stringent safeguards in place to protect the patient’s medical information, as with all forms of digital storage or transfer of sensitive healthcare information. Data security is thus regarded critical for protecting patient privacy [2]. There are multiple ways by which digital data may be accessed by third parties [13], not just for telehealth yet for the digital storage of any information, including data from monitoring devices mentioned previously for example. These include the hacking of data sent over the internet or via Bluetooth, the legal seizing of data by governmental bodies (such as use of subpoena) and access by telecommunication companies and by cloud storage providers, including Internet service provider (ISP) and Google, who may hold ownership of data transmitted through their systems [13, 15]. This risk is exacerbated by the potential for telecommunication companies to then sell on the data they transfer. There is another less complex way third parties may inadvertently become aware of the individual’s health information- simply glancing at the individual’s phone. The security settings on the smartphone, app or device must also be sufficient to ensure the data cannot be accessed readily following theft of the device. It is of great concern that a study found that some mHealth apps do not even use any encryption when transferring data [15]. Patients must be made aware of these risks for consent of data sharing to be valid, and they must also be informed what will happen with their data if they later decide to leave such a scheme [13]. Patients must also be offered the highest possible degree of choice regarding which aspects of their data are shared. Furthermore, there must also be discussion on whether the patient would like to be informed of any incidental findings discovered in the analysis of the constant recordings of their physiological parameters [13]. The complexity of the consent process is heightened further when mobile phones are used to record conversations or locations, for example to examine the impact of Parkinson’s Disease on patients’ speech or their socialisation [16, 17], as third parties who are in conversation with or meeting the patients are inadvertently having their privacy infringed upon, without consent.

There are additional concerns that telehealth may alter the doctor patient dynamic [2]. The ability for healthcare professionals to scrutinise the compliance of patients to treatment regimens leads to a shift in power towards the healthcare provider in any consultation [18]. It also questions whether the patient’s right to privacy is being too readily forfeited. This fear may be partially alleviated if sufficient consent processes are established, as mentioned previously. Another risk is that the use of telehealth may further exacerbate social isolation of the elderly, which may occur if PHM reduces the frequency of visits made by human carers to patients [19].

### Enabling access to digital patient medical records

Immediate access to patient medical records (PMRs) is another arm of constant health monitoring. Sweden has bold plans to enable electronic access to medical records for all citizens over the age of 16 by 2020 [20]. The United Kingdom is following suit, with the NHS also striving to become paperless and offer digital access to patient files. These schemes may enhance the level of patient education and engagement with their condition, and grant further autonomy to the patient.

Providing access to PMRs is dogged with the same hurdles of data security which have been discussed previously in this article. Similar consent processes with opt-in or opt-out options must be established to ensure patients are informed of the relevant risks of this data dissemination and have the option to accept or reject them. There is the additional concern however, that patients will be provided with an undue amount of information, without any training or education on how to interpret it. The consequences of misinterpretation may be significant, with this potentially not only altering patients’ compliance with treatment regimens, yet it may also have a psychological impact on the individuals. Guidance ought therefore to be issued for patients to help them understand the contents of their medical records, or else the transfer of autonomy to the patient is fictitious. The risk of misinterpretation is even greater if instant access to PMRs included access to test results prior to consultations. It may also expound misinformation, if patients are drawn to ill-informed websites in an attempt to understand what their test results mean when lacking the advice of an experienced medical professional. This potential outcome has generated concern, particularly among some clinicians [21]. There has been some studies to investigate the evidence of this assumption, and interestingly one study of cancer patients’ attitudes found that patients would prefer to find out results before consultations [22], with other studies finding no increase in anxiety following access to PMRs [23, 24]. Nonetheless, to reduce the risks of misinformation and misinterpretation, there must be a resolute effort to educate patients on PMRs prior to the enabling of full and instant access.

### Wearable devices

Wearable devices are diverse in function, the breadth of which was too expansive for the scope of this debate. There are two areas which particularly warrant in depth discussion. Firstly, whether fitness tracking wearable devices are effective in driving lifestyle changes, and the ethical implications for wearable devices such as fall monitors for elderly individuals. These areas are quite distinct, yet illustrate how practical and ethical considerations are both critical for evaluating any such device.

Wearable devices on the general market claim to encourage individuals to make lifestyle changes. At the time of writing, the Fitbit® website asks the visitor to “See how Fitbit can help you exercise, eat, sleep and live better” [25] with the assumption being that gaining data on these factors will help people strive for their personal health goals. Two key studies have analysed whether fitness trackers are able to deliver the lifestyle improvements they claim. A study in Singapore by Finkelstein et al., analysing the health effects of the Fitbit Zip® noticed a decline in usage of the device over the study period, with 40% of people stopping usage within 6 months and 90% by a year [26]. Before too readily drawing conclusions about wearable devices from this, this trial used an upper-arm sensor device which was discontinued in 2014, differing significantly from current wrist-worn models [27], highlighting a problem in the methodology of clinical trials for such devices, as they may be outdated and no longer accurately reflect the field at the time of publishing. The users of fitness trackers in the study did appear to have higher levels of activity after a year of use, yet the effect of this on health is questionable as the study found that the Fitbit® wearers did not have improved health outcomes regarding weight, blood pressure or heart rate. This was not an overly large study, with only around 200 people in each intervention and control group, yet it nonetheless fails to support that fitness trackers will lead to improved health outcomes. Another study (the IDEA clinical trial by Jakicic et al.,) was more controversial, suggesting that fitness trackers may be damaging [28]. The effect of standard weight loss schemes was compared with the same schemes enhanced with a wearable device which provided the user with feedback on their physical activity levels. Surprisingly, it found that the group with the added intervention of the wearable device lost less weight over a 24 month time period. To understand why these devices were unsuccessful in clinical trials, there is a requirement for the field to investigate in more detail what the proposed ‘mechanisms of action’ for lifestyle modifying wearable devices are. One commonly cited potential mechanism may be through providing feedback on how the individual is meeting health goals, yet its validity requires more detailed investigation. Therefore, at present it is unclear how such wearable devices are proposed to work, and if they have any effect on health at all. More specific studies are required, with one example being an ongoing study investigating whether a doctor or family member supervising the activity levels of a wearable-device user may provide a greater incentive for increasing activity levels [29]. Such studies provide more insight and will enable the field to progress.

The ethical considerations for wearable devices include whether they influence patient autonomy, whether they can lead to stigma, and whether there is an infringement of privacy through ‘covert monitoring’. These concepts may be well illustrated with fall monitors for elderly individuals, utilised principally to enhance user safety. Addressing the first concept, a reliance on PHM may risk an erosion of individual autonomy [2]. To clarify with an example, elderly patients dependent on fall monitors may become less self-reliant, and with this there may be a reduced sense of self-determination or ability to choose to take risks [2]. Secondly, wearing any device may alter the individual’s perception of themselves, or others’ perception of them. A group study of elderly individuals in residential care found that participants were concerned that using technological devices to assist with frailty would lead to a perception of them being frail [30]. The development of this stigma may be a result of wearing an embodiment of the illness [2]. This need not be visible however; their perception of the stigma may lead to the individual altering their self-identity, even if the wearable device is concealed. If they consider themselves to be frail for example, they may then self-impose pressure to act according to their image of frailty [2]. Thirdly, the concept of covert monitoring must be addressed. The presence of monitoring devices in the homes of individuals may be forgotten over time. This would be particularly prominent when the individual concerned has dementia. Monitoring an individual who is unaware it is occurring raises concerns regarding consent [31], which may extend to inadvertently monitoring guests at the home of an individual with monitoring equipment. These three ethical concerns are substantial and cannot simply be dismissed in the pursuit of greater safety for individuals.

## Conclusion

“Doing all those boring things you do to stay healthy may or may not make you live longer. However, I am sure of one thing; it will make your life seem longer” [32]. This may no longer be the case with technological advances simplifying self-monitoring and collating all the data in one place, the smartphone. These measures will greatly assist patients with chronic diseases who already have to self-manage their condition daily, for the rest of their lives. We must hold fast however to ensure that the rights of citizens are not violated in the pursuit of less troublesome disease management. Data security protocols can be poorly adhered to within the NHS [33], the consent processes for data sharing are in their infancy and the effect of PHM on the individual and their home environment is inadequately established. These technological systems cannot ethically be permitted if they require forfeit of the privacy of patient data by transferring it across insecure data networks. These risks are too evident for policy makers to feign ignorance of this Orwellian assault on civil liberties. We cannot allow the monitoring of health to lead to a situation where patient health records, treatment and even their whereabouts are held ownership by tech-giants, telecommunication companies, the state, or are freely available for all interested parties. Any introduction of medical devices into healthcare must be matched with increased patient education as to their benefits and risks. Their effectiveness must be established, as occurs for all medical treatments, and addressing ethical concerns should be further integrated into the development process of these devices. Yes, technological advancement may well enhance patient care, yet they will harm the identity of the individual if these shortfalls are not addressed.

### About the debate

Following persuasive arguments from both sides, the panel of judges declared the Oxford team (opposition) victorious. The Cambridge team focused their argument on the risks of medicalisation, the exacerbation of health inequalities and the potential for patient misinterpretation of PMRs and data collected by monitoring devices. The Oxford team countered these points and further highlighted how constant health monitoring may increase patient safety through enhancing compliance with treatment regimens and identifying patient deterioration faster. The Oxford team emphasised how these may reduce hospital admissions and improve patient care. Most contention arose on the app Natural Cycles®, and whether it is beneficial for women as a side-effect free contraceptive option, or if instead it is a risk by advertising itself on a platform apart from medical professionals. Following the debate, the teams resolved this by agreeing that women should be offered the option of Natural Cycles®, yet clear guidance should be issued from health bodies, placing it in context alongside the other contraceptive options. This is a note which resonated throughout our discussions, that the risks of medical devices must be transparent for patients and healthcare bodies, and that there is an onus on device developers and regulatory bodies to protect patients’ rights of safety, consent and privacy.

## Abbreviations

AF: Atrial fibrillation
CGM: Continuous glucose monitoring
COPD: Chronic obstructive pulmonary disorder
ECG: Electrocardiogram
mHealth: Mobile health
NHS: National Health Service
NICE: National institute for health and care excellence
PHM: Personal health monitoring
PMR: Patient medical record
UK: United Kingdom of Great Briton and Northern Ireland
